# On the Varieties of Headache

**Published:** 1847-09

**Authors:** 


*On the Varieties of Headache.*—The various circumstances under which headache may arise as a prominent symptom, are thus briefly explained by Dr. Wright in a series of clinical lectures.

To give you a general notion of them, as we are yet dealing in generalities—suppose a patient comes to you complaining of headache. This is a very generic sort of term, and may involve a great variety of specialities, some serious, and others simple. One patient, we will say, is in his teens, or not far out of them, yet he looks older by many years than he ought to do. His face is blanched and parchment-like, cheeks sunken, eyes hollow, lustreless, and watery, and they never look fairly at you; the man is timid, nervous, shuns society, and has no inclination for active pursuits; he is subject to giddiness and forgetfulness, and has almost constantly a dull, heavy pain at the back of his head, perhaps extending down the spine, with a sense of weight and dragging of his legs. Here you have a nervous system enfeebled and shaken from causes you will easily learn if you pointedly inquire after the personal habits of the sufferer. Another complains of oppressive pain chiefly over his eyes, scarcely ever leaving him, but distressingly aggravated at different periods of the day. It is probable, that these periods are subsequent to meal times, and that then the headache is attended also with drowsiness. The man is dyspeptic. He will tell you that his bowels are confined, and that he is troubled with wind. Look at his tongue, and you will see that it is furred with, most likely, a brownish patch in the centre. Percuss the right hypochondrium, and you may find a greater extent of dullness, or more tenderness, than natural. The condition of the great viscus here is wrong. Liver, stomach. and bowels, are the sources of that frontal headache. Another patient has pain in the forehead, but it is acute and lancinating, and not persistent. Its periods of accession and departure are pretty regular. Ask the precise spot of the pain, and you will have indicated the exit of the supra-orbital nerve of either side, probably the left Here you have a case of tic douloureux, which may have no obvious exciting cause, or may result from exposure to cold, from dyspepsia, from pregnancy, from uterine disease or disorder, &c. Another complains of aching all over his head, considerably increased by heat or cold, as the case may be. On further inquiry, you learn that the pain is chiefly superficial, and that to rub the patient's hair in different directions, sharply, is to agonize it. Here you have rheumatism of the cranial integuments. Look cautiously after this case. You may suddenly have a pain of a different kind, and deeper-seated, ushered in by screaming and shouting, followed by restlessness and delirium, with a glaring injected eye—the meninges of the brain will be suffering from metastatic rheumatism in its most active form. It was gout, thus transferred, that destroyed the valuable life of Dr. Ingleby, your late Professor of Midwifery. Another has acute pain darting through his temples and ears, especially when he gets warm in bed; at the same time, he has what he well describes as 'gnawing pains' in his shin-bones; his nose is tender, and the roof of it painful; he has, or has had, sore throat, and there are copper-colored patches about his body. This headache has its foundation in syphilis; mind your treatment, or the more delicate bones of the head and face may be sacrificed.

A delicate female complains of heavy throbbing pain over the middle, or at the back of the head. She has had it several months, more or less, and is liable to periodical exacerbations. The uterus has likely something to do with this pain. It may be a case of simple amenorrhœa; it may denote the climacteric period of female life; it may depend upon pregnancy; or the uterus may be undergoing some morbid change. This organ, however, may not be at fault; habitual constipation, which females are often in the habit of neglecting, may be the cause of the suffering, or it may be occasionally by hemorrhoids.

Such, and so many, nay, many more, are the varieties of pains in the head, having different causes, and requiring different forms of treatment.—*Medical Times.*—*Ibid.*

